# 肿瘤治疗相关骨髓增生异常综合征和急性髓系白血病的临床特征及预后

**DOI:** 10.3760/cma.j.issn.0253-2727.2023.09.007

**Published:** 2023-09

**Authors:** 旭升 徐, 虹 丁, 昕 张, 益 廖, 赫 李, 钦瑜 刘, 家卓 刘, 丽 张, 杰 黄, 玉萍 龚, 洪兵 马, 兵 向, 阳 代, 理 侯, 晓 帅, 挺 牛, 俣 吴

**Affiliations:** 1 四川大学华西医院血液内科，成都 610041 Department of Hematology, West China Hospital, Sichuan University, Chengdu 610041, China; 2 九江市第一人民医院血液内科，九江 332000 Department of Hematology, Jiujiang First People's Hospital, Jiujiang 332000, China

**Keywords:** 肿瘤, 治疗相关急性髓系白血病, 骨髓增生异常综合征, Tumor, Therapy-related acute myeloid leukemia, Myelodysplastic syndrome

## Abstract

**目的:**

探讨肿瘤治疗相关骨髓增生异常综合征和急性髓系白血病（t-MDS/AML）的临床特征、分子生物学特点、疗效以及预后。

**方法:**

回顾性分析2010年1月至2023年4月四川大学华西医院86例肿瘤t-MDS/AML患者临床资料，分析肿瘤t-MDS/AML患者的临床特征、分子生物学特点、治疗及其生存情况。

**结果:**

研究共纳入86例肿瘤t-MDS/AML患者，原发肿瘤类型主要包括乳腺癌（27.9％）、肠癌（17.4％）、淋巴瘤（12.8％）、肺癌（11.6％）。t-AML患者67例，其中M_0_ 1例、M_1_ 6例、M_2_ 27例、M_3_ 9例、M_4_ 12例、M_5_ 10例、M_6_ 1例、M_7_ 1例。62例患者可进行遗传学分层，20例（29.9％）低危组患者中位总生存（OS）时间为36（95％*CI* 22～52）个月；10例（14.9％）中危组患者中位OS时间为6（95％*CI* 3～9）个月；32例（47.8％）高危患者中位OS时间为8（95％*CI* 1～15）个月。非低危组t-AML患者中位OS时间为8（95％*CI* 3～13）个月，明显短于低危组（*χ*^2^＝13.856，*P*<0.001）；非急性早幼粒细胞白血病（APL）的t-AML低危组患者中位OS时间为27（95％*CI* 18～36）个月，长于非低危组（Breslow，*χ*^2^＝5.534，*P*＝0.019），尤其早期OS率差异显著。9例APL病例均按照原发初治APL诱导维持治疗，中位OS时间未达到，1、2、3年OS率分别为100.0％、（75.0±6.2）％、（75.0±6.2）％。58例非APL的t-AML患者中，52例（89.7％）接受化疗，首次诱导化疗完全缓解16例（30.8％）。非APL的t-AML患者的1、2、3年OS率分别为（42.0±6.6）％、（22.9±5.7）％、（13.4±4.7）％。经化疗达到骨髓缓解的患者中位OS时间显著长于未能缓解的患者24（95％*CI* 18～30）个月对6（95％*CI* 3～9）个月，（*χ*^2^＝6.087，*P*＝0.014）。13例患者使用含维奈克拉方案化疗骨髓CR 7例（53.8％），中位OS时间为12（95％*CI* 9～15）个月，和不含维奈克拉方案化疗对比差异无统计学意义（*χ*^2^＝2.343，*P*＝0.126）。19例t-MDS患者中，1、2、3年OS率分别为（46.8±11.6）％、（17.5±9.1）％、（11.7±9.1）％，中位OS时间为12（95％*CI* 7～17）个月，与t-AML相比差异无统计学意义（*χ*^2^＝0.656，*P*＝0.418）。

**结论:**

临床上t-MDS/AML的原发肿瘤为乳腺癌、肠癌等较为常见，具有较高的不良遗传学风险。其中APL患者的诱导缓解率高、长期预后较好，非APL患者缓解率低且预后不良。

## Abstract (en)

**Objective:**

To investigate the clinical characteristics, cytogenetics, molecular biology, treatment, and prognosis of patients with therapy-related myelodysplastic syndrome and acute myeloid leukemia (t-MDS/AML) secondary to malignancies.

**Methods:**

The clinical data of 86 patients with t-MDS/AML in West China Hospital of Sichuan University between January 2010 and April 2023 were retrospectively analyzed. The clinical characteristics, primary tumor types, and tumor-related therapies were analyzed.

**Results:**

The study enrolled a total of 86 patients with t-MDS/AML, including 67 patients with t-AML, including 1 patient with M_0_, 6 with M_1_, 27 with M_2_, 9 with M_3_, 12 with M_4_, 10 with M_5_, 1 with M_6_, and 1 with M_7_. Sixty-two patients could be genetically stratified, with a median overall survival（OS）of 36（95％ *CI* 22–52）months for 20（29.9％）patients in the low-risk group and 6（95％ *CI* 3–9）months for 10（14.9％）in the intermediate-risk group. The median OS time was 8（95％ *CI* 1–15）months in 32（47.8％）patients in the high-risk group. For patients with non-acute promyelocytic leukemia（APL）and AML, the median OS of the low-risk group was 27（95％ *CI* 18–36）months, which was significantly longer than that of the non-low-risk group（*χ*^2^＝5.534, *P*＝0.019）. All 9 APL cases were treated according to the initial treatment, and the median OS was not reached, and the 1-, 2-, and 3-year OS rates were 100.0％,（75.0±6.2）％, and（75.0±6.2）％ respectively. Of the 58 patients with non-APL t-AML（89.7％）, 52 received chemotherapy, and 16 achieved complete remission（30.8％）after the first induction chemotherapy. The 1-, 2-, and 3-year OS rates of the non-APL t-AML group were（42.0 ± 6.6）％,（22.9±5.7）％, and（13.4±4.7）％, respectively. The median OS of patients who achieved remission was 24（95％ *CI* 18–30）months, and the median OS of those who did not achieve remission was 6（95％ *CI* 3–9）months（*χ*^2^＝10.170, *P*＝0.001）. Bone marrow CR was achieved in 7（53.8％）of 13 patients treated with vineclar-containing chemotherapy, with a median OS of 12（95％ *CI* 9–15）months, which was not significantly different from that of vineclar-containing chemotherapy（*χ*^2^＝0.600, *P*＝0.437）. In 19 patients with t-MDS, the 1-, 2-, and 3-year OS rates were（46.8±11.6）％,（17.5±9.1）％, and（11.7±9.1）％ with a median OS of 12（95％ *CI* 7–17）months, which was not significantly different from that in t-AML（*χ*^2^＝0.232, *P*＝0.630）.

**Conclusion:**

Breast cancer, bowel cancer, and other primary tumors are common in patients with t-MDS/AML, which have a higher risk of adverse genetics. Patients with APL had a high induction remission rate and a good long-term prognosis, whereas patients without APL had a low remission rate and a poor long-term prognosis.

肿瘤治疗相关骨髓增生异常综合征及急性髓系白血病（t-MDS/AML）指接受了肿瘤放疗或辅助化疗后发生的MDS和AML，属于WHO 2016年修订的髓系肿瘤分类中的一个亚型[1]。随着诊疗技术的进步、新药的使用，肿瘤患者生存时间延长，t-MDS/AML在临床上发生率也逐渐增高[2]–[3]。多项研究表明t-MDS/AML普遍预后不良，总生存（OS）率低，只有10％～17％的t-AML患者在诊断后5年仍存活[4]–[5]。本研究我们回顾性分析了四川大学华西医院86例肿瘤t-MDS/AML患者的临床数据及预后结局，以进一步探讨t-MDS/AML患者的临床及预后特点。

## 病例与方法

一、病例资料

回顾性收集2010年1月至2023年4月于四川大学华西医院诊疗的86例t-MDS/AML患者的临床资料，包括性别、年龄、原发肿瘤类型、诊断t-MDS/AML的年龄、两次肿瘤间隔时间。复习纳入病例的骨髓形态学、免疫分型、细胞遗传学、分子生物学等诊断信息，t-MDS/AML的治疗方案和结局等。诊断、预后分层以及疗效评估标准参照《中国成人急性髓系白血病（非急性早幼粒细胞白血病）诊疗指南（2021年版）》[6]、《中国急性早幼粒细胞白血病诊疗指南（2018年）》[7]及《骨髓增生异常综合征中国诊断与治疗指南（2019年）》[8]。

二、分子生物学检测

融合基因检查包含AML1/ETO、CBFβ/MYH11、PML/RARa、BCR/ABL、EVI1表达（非融合基因，为基因表达高表达，纳入融合基因panel报告）、MLL重排和dup-MLL、Nup98重排；预后基因至少为34个基因panel，包括：ASXLI、BCOR、BCORL1、CALR、CBL、CEBPA、CSF3R、DNMT3A、ETV6、ETNK1、EZH2、FLT3、IDH1、IDH2、JAK2、KIT、KRAS、MPL、NF1、NPM1、NRAS、PHF6、PIGA、PTPN11、RUNX1、SETBP1、SF3B1、SRSF2、STAG2、TET2、TP53、U2AF1、WT1、ZRSR2。

三、生存指标定义及随访

OS时间定义为确诊t-MDS/AML至死亡或随访截止时间。采用查阅患者门诊/住院病历和电话进行随访，随访截止时间为2023年4月，中位随访62（95％ *CI* 46～78）个月。

四、统计学处理

采用SPSS 26.0软件进行统计学分析，R 4.2.2绘制热图。非正态分布连续变量用中位数（*Q*_1_，*Q*_3_）表示，组间比较采用Mann-Whitney *U*检验；分类变量用例数（百分比）表示，组间比较采用卡方检验或Fisher精确概率法；采用Kaplan-Meier法绘制生存曲线，组间比较采用Log-rank或Breslow检验。*P*<0.05为差异有统计学意义。

## 结果

一、患者临床特征

本研究共收集86例患者，男36例（41.9％），原发肿瘤距诊断t-MDS/AML中位时间48（24，72）个月，t-MDS/AML诊断时中位年龄57.5（49，67）岁，诊断t-MDS/AML时中位WBC为 4.0（1.6，15.7）×10^9^/L、中位HGB为77（62，97）g/L、中位PLT为36（18，70）×10^9^/L。其中t-AML 67例，t-MDS 19例，t-AML和t-MDS相比，原发肿瘤距诊断t-MDS/AML时间、t-MDS/AML诊断时年龄、诊断t-MDS/AML时的血常规指标差异均无统计学意义，临床特征详见表1。

**表1:** 肿瘤治疗相关骨髓增生异常综合征/急性髓系白血病（t-MDS/AML）患者的一般临床特征

临床特征	总体（86例）	t-AML（67例）	t-MDS（19例）	统计量	*P*值
性别[例（%）]				Fisher	0.016
男	36（41.9）	23（26.7）	13（15.1）		
女	50（58.1）	44（51.2）	6（6.9）		
原发肿瘤距诊断t-MDS/AML时间[月，*M*（*Q1, Q3*）]	48（24, 72）	36（24, 60）	60（36, 108）	2.458	0.121
t-MDS/AML诊断时年龄[岁，*M*（*Q1, Q3*）]	57.5（49, 67）	56（47, 66）	59（50, 70）	−1.391	0.164
诊断t-MDS/AML时WBC[×10^9^/L，*M*（*Q1, Q3*）]	4.0（1.6, 15.7）	4.3（2.1, 26.4）	2.5（1.5, 5.2）	−1.804	0.071
诊断t-MDS/AML时HGB[g/L，*M*（*Q1, Q3*）]	77（62, 97）	81（62, 98）	64（57, 83）	−1.630	0.103
诊断t-MDS/AML时PLT[×10^9^/L，*M*（*Q1, Q3*）]	36（18, 70）	38（17, 69）	35（22, 88）	−0.338	0.736
原发肿瘤治疗[例（%）]				1.168	0.558
单纯化疗	41（47.7）	30（34.9）	11（12.8）		
单纯放疗	9（10.4）	7（8.1）	2（2.3）		
化疗+放疗	36（41.9）	30（34.9）	6（7.0）		

原发肿瘤类型包括乳腺癌、肠癌、肺癌、淋巴瘤、甲状腺癌等，由于患者原发肿瘤半数以上为其他医院收治且时间跨度较长，故化疗、放疗剂量和疗程未能详细记载，化疗患者有使用细胞毒性药物，如烷化剂、铂类药物、抗代谢药物、拓扑异构酶Ⅱ抑制剂等，其分布及主要治疗方案详见表2。

**表2:** 86例肿瘤治疗相关骨髓增生异常综合征/急性髓系白血病（t-MDS/AML）患者原发肿瘤分布及治疗情况

原发肿瘤	例数	构成比（%）	主要化疗药物及方案	放疗情况
乳腺癌	24	27.9	AC、TAC、TC、TX等	16例联合放疗
肠癌	15	17.4	X、F、L-OHP等	7例联合放疗、1例单用放疗
淋巴瘤	11	12.8	ABVD、CHOP	3例联合放疗
肺癌	10	11.6	EP、TP、GP等	4例联合放疗、1例单用放疗
膀胱癌	4	4.7	CPT、MMC、P	无
甲状腺癌	4	4.7	无	4例为碘-131
宫颈癌	3	3.5	TP、P	2例联合放疗
食管癌	3	3.5	TP、TX	2例联合放疗
卵巢癌	2	2.3	TP	1例联合放疗
前列腺癌	2	2.3	无	2例单用放疗
鼻咽癌	1	1.2	PF	1例联合放疗
多发性骨髓瘤	1	1.2	CTD	无
肝癌	1	1.2	FOLFOX	无
高级别胶质瘤	1	1.2	无	1例单用放疗
骨肉瘤	1	1.2	P	无
肾癌	1	1.2	不详	无
输卵管肿瘤	1	1.2	不详	无
急性白血病^a^	1	1.2	DA、HD-AraC、ASCT	无

注 A：蒽环类药物；C：环磷酰胺；T：紫杉类药，含多西他赛、紫杉醇、白蛋白紫杉醇；X：卡培他滨；F：氟尿嘧啶类；L-OHP：奥沙利铂；ABVD：多柔比星+博来霉素+长春新碱+氮烯咪胺；CHOP：环磷酰胺+多柔比星+长春新碱+泼尼松；E：依托泊苷；P：顺铂或卡铂；G：吉西他滨；CPT：喜树碱；MMC：丝裂霉素；CTD：环磷酰胺+沙利度胺+地塞米松；FOLFOX：奥沙利铂+氟尿嘧啶+亚叶酸钙；DA：柔红霉素+阿糖胞苷；HD-AraC：大剂量阿糖胞苷；ASCT：自体造血干细胞移植。^a^患者既往确诊t（8;21）、AML1/ETO融合基因阳性AML，ASCT后融合基因持续阴性，5年后因全血细胞减少诊断为具有−7遗传学改变的t-MDS，其原有的融合基因仍阴性

67例t-AML患者按照FAB分型进行形态学诊断分类，其中M_0_ 1例、M_1_ 6例、M_2_ 27例、M_3_ 9例、M_4_ 12例、M_5_ 10例、M_6_ 1例、M_7_ 1例；62例患者可进行遗传学分层，其中低危20例（29.9％）、中危10例（14.9％）、高危32例（47.8％）。按照WHO 2016分型对19例t-MDS患者进行诊断分型，其中MDS伴原始细胞增多-1（MDS-EB1）5例、MDS伴原始细胞增多−2（MDS-EB2）9例、MDS伴多系病态造血（MDS-MLD）2例、MDS未分类（MDS-U）3例。

二、生物学特征

1. 细胞遗传学特点：60例患者可进行染色体核型分析，其中16例（26.6％）为正常核型，19例（31.7％）有1种染色体异常，7例（11.7％）有2种染色体异常，复杂核型18例（30.0％）。

2. 分子生物学特点：在纳入86例患者中81例有髓系常见融合基因和预后基因突变结果。28例患者融合基因阳性，其中9例急性早幼粒细胞白血病（APL）患者均为经典PML/RARa融合基因。19例非APL患者中7例为AML1/ETO，4例为CBFβ/MYH11，2例同时MLL/AF6融合基因和EVI1阳性，EVI1和MLL/ELL各有2例，NPM/MLF1和MLL/AF9各1例。

髓系基因突变结果显示46例患者检测出至少1个基因突变，30例（37.5％）患者具有2个及以上突变。其中最常见的是TP53突变（14例，17.3％）及ASXL1突变（14例，17.3％），其他主要突变依次有TET2（11例，13.6％）、FLT3-ITD（8例，9.9％）、RUNX1（7例，8.6％）、DNMT3A（7例，8.6％）、WT1（5例，6.2％）。具体突变的情况见图1。

**图1:**
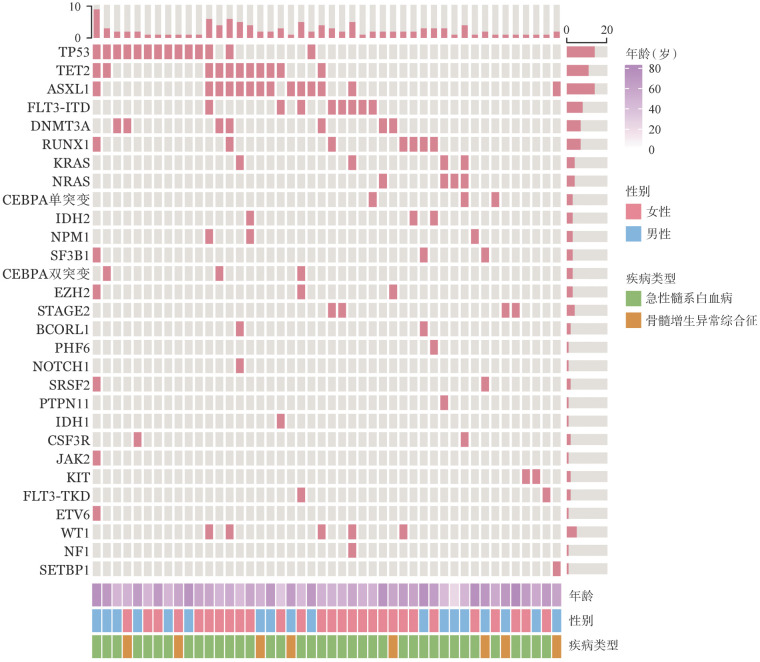
46例肿瘤疗相关骨髓增生异常综合征/急性髓系白血病（t-MDS/AML）患者基因突变图谱

三、治疗和生存情况

67例患者确诊t-AML后，9例APL病例均按照原发初治APL使用亚砷酸联合维A酸诱导维持治疗；58例非APL的t-AML患者中，接受化疗52例（89.7％），化疗药物以去甲基化药物、维奈克拉、DA（柔红霉素+阿糖胞苷）、IA（去甲氧柔红霉素+阿糖胞苷）、HAG（高三尖杉酯碱+阿糖胞苷+G-CSF）、CAG（阿克拉霉素+阿糖胞苷+G-CSF）、阿糖胞苷等为主。DA等传统化疗药物治疗方案有22例，含去甲基化药物、维奈克拉化疗患者有27例，3例患者化疗方案不详，6例选择支持治疗。7例患者化疗缓解后接受了骨髓移植，4例为HLA配型全相合，3例为HLA配型半相合。

67例t-AML患者中位OS时间为12（95％*CI* 6～18）个月，1、2、3年OS率分别为（49.8±6.2）％、（29.9±5.8）％、（21.3±5.3）％。62例可进行遗传学分层患者中，低危组、中危组、高危组中位OS时间分别为36（95％*CI* 22～52）、6（95％*CI* 3～9）、8（95％*CI* 1～15）个月。总体来看，非低危组患者中位OS时间为8（95％*CI* 3～13）个月，明显短于低危组（*χ*^2^＝13.856，*P*<0.001）（图2A）。排除APL后进一步分析，非APL的t-AML患者中的低危组患者中位OS时间为27（95％*CI* 18～36）个月，明显长于非低危组（Breslow，*χ*^2^＝5.534，*P*＝0.019）（图2B）。

APL患者中诱导化疗后全部获得完全缓解（CR），中位OS时间未达到，1、2、3年OS率分别为100.0％、（75.0±6.2）％、（75.0±6.2）％。1例为CR两年后复发死亡，1例为CR达2年后死于新型冠状病毒感染。

非APL的t-AML患者的中位OS时间为11（95％*CI* 7～15）个月，1、2、3年OS率分别为（42.0±6.6）％、（22.9±5.7）％、（13.4±4.7）％。52例化疗患者中位OS时间为12（95％*CI* 8～16）个月，6例支持治疗的患者中位OS时间为4（95％*CI* 0.4～8）个月。首次诱导化疗骨髓达CR 16例（30.8％），中位OS时间为24（95％*CI* 18～30）个月，36例（69.2％）未达到CR患者中位OS时间为 6（95％*CI* 3～9）个月，差异有统计学意义（*χ*^2^＝6.087，*P*＝0.014）（图2C）。传统的DA、IA方案化疗有16例，其中5例（31.3％）骨髓CR，中位OS时间为16（95％*CI* 0～36）个月，3年OS率为（25.0±10.8）％；13例患者使用含维奈克拉方案化疗骨髓CR 7例（53.8％），中位OS时间为12（95％*CI* 9～15）个月，其他不含维奈克拉治疗方案治疗中位OS时间为14（95％*CI* 4～24）个月，维奈克拉治疗未显示生存优势（*χ*^2^＝2.343，*P*＝0.126）（图2D）。7例患者接受骨髓移植，5例获长期缓解，2例疾病复发后死亡。

**图2:**
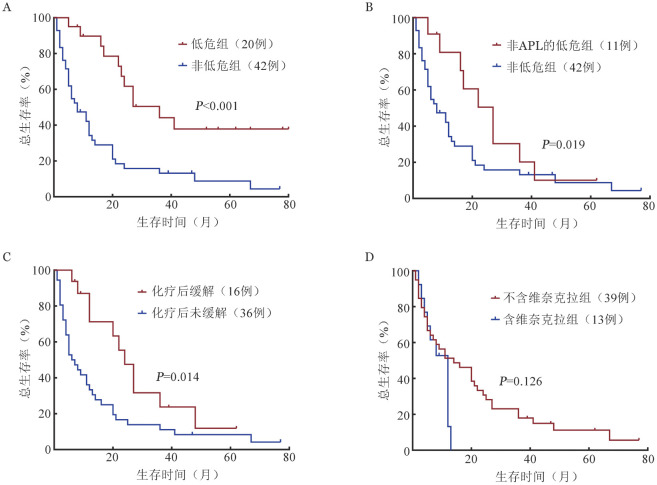
t-AML亚组生存曲线 **A** 低危组与非低危组；**B** 非APL亚组中低危组与非低危组；**C** 化疗未缓解组与化疗缓解组；**D** 不含维奈克拉与含维奈克拉化疗组 注 t-AML：治疗相关急性髓系白血病；APL：急性早幼粒细胞白血病

19例t-MDS患者中，9例接受去甲基化药物或维奈克拉化疗，1例接受小剂量阿糖胞苷化疗，2例接受HAG化疗，余7例为支持治疗。1、2、3年OS率分别为（46.8±11.6）％、（17.5±9.1）％、（11.7±9.1）％，中位OS时间为12（95％*CI* 7～17）个月，与t-AML相比差异无统计学意义（*χ*^2^＝0.656，*P*＝0.418）。2例MDS-MLD患者OS时间大于5年。1例化疗后未CR，行胞妹HLA全相合外周血造血干细胞移植，6个月后疾病复发死亡。

## 讨论

肿瘤继发t-MDS/AML患者一般体能差、常具有高危的遗传学风险、有多种合并症、不能耐受高强度的化疗、预后差，对临床工作是极大挑战。目前，t-MDS/AML的年龄构成、初始肿瘤的类型、从原发肿瘤到继发髓系肿瘤之间间隔时间、具体的分子生物学特点和预后尚缺乏长期数据。本研究我们通过回顾性分析单中心连续13年数据，是近十年单中心报告肿瘤t-MDS/AML的最大队列，能反映中国大型综合医院t-MDS/AML的特点和结局。

本研究中女性患者数量比男性多，因原发恶性肿瘤中乳腺癌占比高达27.9％，其次是消化道肿瘤、肺癌、淋巴瘤，肿瘤类型和国内国外报道大致相同[2],[9]。t-MDS和t-AML相比，原发肿瘤的时间、t-MDS/AML确诊时间差异均无统计学意义，OS时间基本相同，t-MDS通常快速进展为AML[5]，这两个类别之间的结果不存在明显差异。

本研究的86例患者中46例（53.4％）至少存在1个基因突变，TP53基因突变14例（16.3％），低于国外t-MDS/AML的检出率21％～33.3％[10]–[11]。具有不良的细胞遗传学及分子生物学的t-AML共32例（47.8％），t-MDS/AML相比新发患者的复杂核型和TP53突变频率高[11]。我们的研究显示在t-AML中应用ELN危险分层后，能区分患者预后，尤其是APL患者，肿瘤病史对其预后影响较小，与文献[9],[12]的研究结果一致。

本研究中传统的DA、IA方案化疗有16例，其中5例（31.3％）骨髓CR，含维奈克拉方案诱导化疗的患者13例，骨髓CR 7例（53.8％），缓解率高于传统方案。提高t-AML患者首次诱导缓解率，对于这部分患者生存获益至关重要。肿瘤t-AML患者无法耐受高强度化疗，首次诱导缓解率较低，明显低于原发初治患者。伴随目前新药的发展，肿瘤继发t-MDS/AML疗效较前有所改善。很多研究使用阿扎胞苷、维奈克拉、CPX-351（阿糖胞苷和柔红霉素以5∶1的固定摩尔比组成的脂质体制剂）可以提高患者缓解率、延长OS时间。Lancet等[13]进行的随机临床试验研究显示，CPX-351治疗老年高危或继发性AML的CR+血细胞计数未完全恢复的CR（CRi）率为48％，中位OS时间为9.33个月，3年、5年OS率分为21％、18％，CPX-351治疗5年OS率优于传统“7+3”方案。Grenet等[14]的研究显示，阿扎胞苷+维奈克拉方案的总体CR+CRi率、中位无复发生存（RFS）和OS时间分别为56.6％、14.1个月和11.1个月，疗效与CPX-351方案一致。以新药和分子靶向药物为基础的联合诱导方案有望提高这部分患者首次诱导缓解率。

相关研究显示t-AML早期缓解行异基因造血干细胞移植，可以进一步延长患者OS时间[15]–[17]。本研究中8例患者接受移植，5例获得长期生存。因样本量较少，需要多中心和长期随访数据来进行验证。

本研究聚焦中国大型综合三甲医院对肿瘤t-MDS/AML13年数据，结果显示乳腺癌、肺癌和消化道肿瘤患者是t-MDS/AML主要人群。肿瘤t-MDS/AML总体预后仍差。细胞遗传学和分子生物学为基础的危险分层对于判断患者预后有重要意义，t-APL疗效与原发APL相似，非APL的t-AML低危组患者预后较好，缓解患者有条件进行异基因造血干细胞移植可带来长期生存获益。由于新药和免疫治疗的不断涌现，未来需要更大样本和更长期随访的多中心研究进一步探索这类患者的治疗模式。
